# Characterizations of Bacterial Vaginosis among HIV-Positive and HIV-Negative Women in Rural Eastern Cape Province, South Africa

**DOI:** 10.1155/2021/9913878

**Published:** 2021-07-21

**Authors:** Teke Apalata, Sandisiwe Nojaholo, Ikanyeng D. Seipone, Ntombizodumo Nxasana

**Affiliations:** ^1^Division of Medical Microbiology, Department of Laboratory Medicine and Pathology, Faculty of Health Sciences, Walter Sisulu University, Mthatha 5100, South Africa; ^2^National Health Laboratory Services (NHLS), Mthatha 5100, South Africa; ^3^Research Unit, Faculty of Health Sciences, Walter Sisulu University, Mthatha 5100, South Africa

## Abstract

Bacterial vaginosis (BV) is extremely common among the African population and is associated with the transmission and acquisition of human immunodeficiency virus (HIV) infection. The objective of this study was to determine the prevalence and characteristics of BV among HIV-infected and -uninfected women in rural Eastern Cape province of South Africa. A descriptive cross-sectional study was conducted between September 2017 and March 2018 on women aged 18 years and above (*n* = 100), attending Nelson Mandela Academic Hospital and Ngangelizwe Community Health Centre with signs and symptoms suggestive of vaginal infection. High vaginal swabs were collected, and BV was diagnosed using Nugent's score. The prevalence rate of BV was 70% irrespective of HIV status. Of the 61 HIV-infected patients, 49 (80.3%) and 12 (19.7%) were BV positive and BV negative, respectively; whilst of the 39 HIV-uninfected women, 21 (53.8%) and 18 (46.2%) were BV positive and BV negative, respectively (OR = 3.5; CI: 1.4–8.5; *p*=0.005). Women aged above 35 years were highly likely to develop BV (*p*=0.049). The presence of *Mobiluncus* species (>25 per high microscopic field) was significantly associated with BV among HIV-infected patients (*p*=0.030). A recent history of antibiotic use (≤3 months) was significantly associated with BV among HIV-negative patients (*p*=0.044). This study shows that BV is more prevalent among HIV-positive women than their HIV-negative counterparts, and its occurrence is higher among those aged above 35 years. The predominance of *Mobiluncus* species in the vagina microbiota of HIV-infected women might play a significant role in the development of BV. These findings suggest that the treatment of BV could restore normal flora and reduce susceptibility to and transmission of HIV.

## 1. Introduction

Bacterial vaginosis (BV) is the most common genital infection worldwide with reported high prevalence among the African population [1, 2]. It has been associated with the transmission and acquisition of human immunodeficiency virus (HIV) infection, which poses a major problem for resource-limited Sub-Saharan Africa that bears more than 70% of the global burden of HIV infections with women accounting for 58% of the total number of people living with HIV [1, 3]. BV is a condition that occurs due to the disruption of a healthy vaginal flora, resulting in an overgrowth of Gram-negative anaerobic bacteria, and the condition may be accompanied by symptoms such as discharge, itching, and pain in patients [4].

Although the etiology of BV is complex and controversial, it is often associated with the absence or decrease of protective lactobacilli that are normally present in the vagina [5, 6]. Lactobacilli maintains the vaginal acidity or pH by producing lactic acid from glycogen, which inhibits the growth of the low level bacterial species found in the vagina such as *Mobiluncus*, *Gardnerella,* and *Bacteroides*. Certain *Lactobacillus* species also produce hydrogen peroxide which is toxic to bacteria as well as viruses such as HIV [5]. Lack of lactobacilli can lead to overgrowth of bacteria such as *Mycoplasma hominis*, *Gardnerella vaginalis*, *Provotella*, *Bacteroides*, *Peptostreptococcus,* and other *Mobiluncus* species [6]. Pathogenesis of BV is associated with factors that disrupt the normal acidity of the vagina and the balance in the vaginal microflora. Douching, smoking, using of bubble bath, history of sexually transmitted infections (STIs), and high frequency of sexual intercourse are considered major factors playing a role in the pathogenesis of BV [7].

Women with BV are at higher risk of acquiring sexually transmitted infections (STIs) including HIV [8]. BV is associated with a 60% increased risk of HIV-1 acquisition in women [1, 9]. This has been attributed to changes in the vaginal microflora that occur during BV infection, resulting in a decrease in the number of hydrogen peroxide-producing lactobacilli and an increase in the vaginal pH. This abnormal vaginal microflora cascades an inflammatory response leading to recruitment of immune response cells that become the target cells for the HIV infection in the vagina [10]. Epithelial trauma also occurs during the inflammatory process, allowing an easy access through the epithelium for the virus. These changes are known to provide a conducive environment for HIV replication and survival [10]. It is reported that 50% of HIV-infected women in Africa have BV [1, 9]. Women with BV are also at higher risk of transmitting HIV to their partners. BV was associated with a greater than 3-fold increased risk of female-to-male HIV-1 transmission from BV-positive women in Africa [1].

South Africa bears the heaviest burden of HIV infection in the resource-limited settings of Sub-Saharan Africa, and BV plays a significant role in the acquisition and transmission of HIV. Considerable steps towards reducing the prevalence of BV in Africa can assist in decreasing the number of HIV infections. Current knowledge regarding the association between HIV and BV is still scarce. The present study sought to investigate the association between BV and HIV infection and other well-known risk factors. In addition, this aimed at adding further knowledge on the association between BV and HIV infection in Umtata. The objectives of this study were (i) to measure the prevalence of BV in HIV-positive and -negative women in Umtata healthcare centers, (ii) to determine the association between BV and HIV infection, and (iii) to determine the association between disrupted vaginal microflora and BV.

## 2. Materials and Methods

### 2.1. Ethical Considerations

The current study was approved by the Walter Sisulu University Research Ethics Committee (protocol no. 096/2017). Permissions were granted by the Nelson Mandela Academic Hospital and Ngangelizwe CHC clinical governances. Participants were provided with information about the study and notified that participation was voluntary and were assured of confidentiality of their information by using corresponding study numbers. Informed written consents were obtained from the participants before the commencing of the study.

### 2.2. Study Participants

The study population consisted of 100 women aged 18–50 years who presented with signs and symptoms suggestive of vaginal infection at the Nelson Mandela Academic Hospital and Ngangelizwe Clinic in Mthatha, Eastern Cape province, from September 2017 to March 2018. Informed written consents were obtained from all the participants (see data S1 in the Supplementary Materials for a detailed informed consent). At study entry, a standardised questionnaire was used to collect demographic, gynaecological, medical data as well as behavioural risk factors associated with BV from the participants (see data S2 in the Supplementary Materials for detailed contents of the questionnaire). Demographic data consisted of age, occupation, level of education, and marital status. Gynaecological characteristics included history of gynaecological infections in the past. Behavioural risks included use of contraceptives and use of condoms, whereas medical characteristics included HIV status from available clinical records. Women who did not sign the consent forms, those who were below 18 years of age, and those without HIV results were excluded from the study. Vaginal ProbeTec swabs (Becton Dickinson, Sparks, Maryland, USA) were used to collect genital specimens from patients with vaginal discharge syndrome. These swabs, which collected genital discharge materials from women's posterior fornix, were transported to laboratory and used to make smears onto glass slides for Gram staining procedure.

### 2.3. Laboratory Procedures

#### 2.3.1. Gram Stain

The vaginal swabs collected at enrolment were streaked on sterile examination glass to obtain a smear and heat fixed. The slides were Gram stained according to Schwebke et al.'s [11] techniques and evaluated microscopically under oil immersion at a 100X magnification. Large Gram-positive rods were identified as *Lactobacillus*, small Gram variable rods as *Gardnerella*, small negative rods as *Bacteroides,* and curved Gram variable as *Mobiluncus*.

#### 2.3.2. Nugent's Score

BV was diagnosed using the Nugent's criteria [12]. *Gardnerella* and *Lactobacillus* were given scores between 0 and 4; however, *Mobiluncus* were only graded from 0–2. The total scores were then calculated and interpreted as follows: 0–3 (normal), 4–6 (intermediate bacterial count), and 7–10 (bacterial vaginosis). Participants were diagnosed as BV positive if they had a score of 7 and above [12].

### 2.4. Statistical Analysis

Samples of 100 women were calculated for analyses. Descriptive analyses were performed to describe demographic, medical, and behavioural factors. IBM SPSS statistics for Windows were used for analyses of data. Continuous variables were expressed as median (range) and category variables were expressed as proportions (%). To determine whether there was any significant difference between the observed proportions, a chi-square test was used with a *p* value of <0.05 considered as significant.

## 3. Results

### 3.1. Participants' Demographics and BV Status

A hundred participants (age range is 18 to 50) with a median age of 27.6 years were enrolled. Among the 100 participants, 29% were BV free (Nugent's score: 0–3), 40% were BV intermediate (Nugent's score: 4–6), and 31% were true BV positive (Nugent's score: 7–10). Women aged above 35 years were highly likely to develop BV as compared to those below 35 years (*p*=0.005). Traditional factors (use of antibiotics, douching, diabetes mellitus, and sexually transmitted infections) commonly associated with BV were not found statistically significant impactors in this study (*p* > 0.05) (Table 1).

### 3.2. Analysis of the Association of BV Status and HIV Status

**Figure 1:**
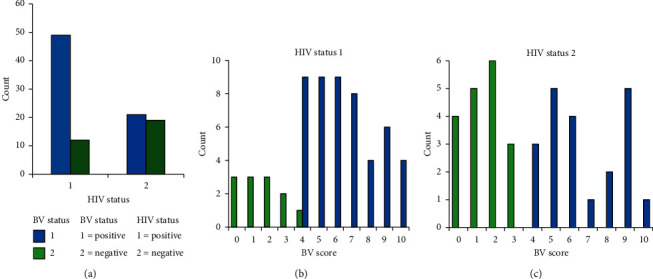
The association between (a) HIV status and BV status, (b) HIV (+) and BV (positive and negative), and (c) HIV (−) and BV (negative and positive).

While 61% of the participants were HIV positive, the remaining 39% were HIV negative. Among the 61 HIV-positive participants, 49 (80.3%) were BV positive and 12 (19.7%) were BV negative. On the other hand, of the 39 HIV-negative participants, 21 (53.8%) were BV positive and 18 (46.2%) were BV negative. The presence of BV was significantly associated with HIV positivity (*p*=0.005), suggesting that there is an association between BV and HIV transmission (Table 1). Majority of HIV-positive women had Intermediate *4*–6 BV score followed by Severe BV 7–10, whereas majority of HIV-negative women had a BV score of 0–3 (Figure 1). Severe BV was prominently recorded in 22 HIV-positive women (71.0%), whereas only 9 (29.0%) HIV-negative women had severe BV (Table 2).

### 3.3. Analysis of Association of BV Species and HIV Infection

The presence of *Mobiluncus* species (>25 in total on high microscopic fields) was significantly associated (*p*=0.030) with BV among HIV-infected patients. *Lactobacillus* was found to be more dominant (*p*=0.000) in HIV-negative while *Mobiluncus* was more dominant in HIV-positive patients. The presence of *Gardnerella vaginalis* was not found to be significantly associated (*p* > 0.05) with BV among the participants irrespective of their HIV status (Table 3).

## 4. Discussion

Our study showed that BV was associated with an increased risk of HIV infection among women older than 35 years of age. Women who are BV positive are reported to be more likely to seroconvert than women who are BV negative [2]. This has been attributed to the physiological changes associated with BV, including vaginal pH changes due to the lack of acid production by hydrogen peroxide-producing lactobacilli, leading to an elevated pH conducive for growth and survival of the HIV virus, and changes on the integrity and permeability of the cervico-vaginal epithelium due to inflammatory reaction in the female genital tract, and changes in the genital microflora that occur with BV [2]. One study showed that women with BV had 2.5-fold increased risk of acquiring HIV [2]. A study in Malawi also reported an HIV incidence rate of 4.51 per 100 person-years after follow-up (95% CI: 2.96–6.06) among BV-positive women of reproductive age [13]. Several studies have shown that women of childbearing age are affected by BV, especially in Sub-Saharan Africa [14, 15].

Many HIV-1-infected women were found to have depleted vaginal lactobacillus (Table 3) which may lead to severe BV, characterized by the decrease of *Lactobacillus* and increase in the proportion of *Mobiluncus* spp. The presence of *Mobiluncus* species (>25 in total on high microscopic fields) was significantly associated with BV among HIV-infected patients in our present study, suggesting the role of this microorganism in causing BV among HIV- positive patients. *Lactobacillus* was found to be more dominant in HIV-negative women while *Mobiluncus* was more dominant in HIV-positive women. *Mobiluncus* is one of the bacterial species recently described as associated with BV [16]. Previous studies suggest that the most predominant bacterial species due to the absence of *Lactobacillus* is *Gardnerella vaginalis* [17]. However, a Ugandan study on women of reproductive age observed no association between BV and *Gardnerella* but reported association between BV and *Mobiluncus* spp. [18]. Another Ugandan study reported that *Mobiluncus* spp. was detected in 84.5% of women with BV and 38% of women without BV, suggesting that *Mobiluncus* was more common in abnormal flora [16]. The significance in the prevalence of *Mobiluncus* on women with BV suggests that the species could be involved in the pathogenesis of BV. Another study also reported that BV-associated microbiota are linked to increased HIV shedding, and vaginal bacterial community density was diversely higher in women who acquire HIV infection [5]. Differences in the vaginal microbial diversity and concentrations of key bacteria were associated with greater risk of HIV acquisition in women [5].

Previous studies reported up to a 2-fold relative risk for developing BV amongst those who douched regularly [19, 20], and sexual activity in black women was shown to be associated with an increased risk of BV [19]. In our study, the use of douching practices showed no significant association with BV. BV has been linked to various pregnancy complications including preterm labor and delivery [21]. The most consistent association was between preterm delivery and BV, suggesting that BV exacerbates the dissemination of the bacteria from the lower to upper genital tract, or BV may be an indication of microbial colonization of the upper genital tract. Therefore, BV is recognized as a risk factor for the upper genital tract infections in both gynecologic and obstetric patients [22]. Previous studies suggested that BV was inversely associated with gestational age with an OR of 0.008 (95% CI: 0.01–0.42;*p* value 0.003) [23], which is in contrast with the present study, since BV was not associated with gestational age in the current study, and it was more diagnosed in nonpregnant women although the difference was not statistically significant.

## 5. Limitations

This study used the Nugent's criteria for BV analysis, because it is cost-effective, and a routine test used in our resource-limited laboratory, highly sensitive molecular diagnostic methods could have been used for a more thorough detection of BV.

## 6. Conclusions

Bacterial vaginosis was associated with the decrease or absence of protective lactobacilli which are normally present in the vagina. BV is more prevalent among HIV-positive women than their HIV-negative counterparts, and its occurrence is higher among those aged above 35 years. The study supports the understanding that BV could be associated with HIV infection acquisition. *Mobiluncus* species in the vagina microbiota of HIV-infected women might play a significant role in the development of BV. These findings suggest that the treatment of BV could restore normal flora and reduce susceptibility to HIV; however, further clinical studies are necessary to verify this supposition.

## Figures and Tables

**Table 1:** Demographic and clinical characteristics of the study participants.

Study population (*n* = 100)		No. of BV (+) participants	No. of BV (−) participants	*p* value^*∗*^

*Age*				0.049
<35 years	82	54 (65.9%)	28 (34.1%)	
>35 years	18	16 (88.9%)	2 (11.1%)	

*Marital status*				0.493
Married	35	26 (74.3%)	9 (25.7%)	
Single	65	44 (67.7%)	21 (32.3%)	

*Income*				0.437
Yes	28	18 (64.3%)	10 (35.7%)	
No	72	52 (72.2%)	20 (27.8%)	

*Use of contraceptives*				0.242
Yes	38	24 (63.2%)	14 (36.8%)	
No	62	46 (74.2%)	16 (25.8%)	

*Use of bubble bath*				0.533
Yes	98	69 (70.4%)	29 (29.6%)	
No	2	1 (50.0%)	1 (50.0%)	

*No. Of sexual partners*				0.127
One	85	62 (72.9%)	23 (27.1%)	
More than one	15	8 (53.3%)	7 (46.7%)	

*Use of condoms*				0.754
Yes	39	28 (71.8%)	11 (28.2%)	
No	61	42 (68.9%)	19 (31.1%)	

*History of STIs*				0.447
Yes	39	29 (74.4%)	10 (25.6%)	
No	61	41 (53.3%)	20 (46.7%)	

*HIV status*				**0.005**
Positive	61	49 (80.3%)	12 (19.7%)	
Negative	39	21 (53.8%)	18 (46.2%)	

*Use of antibiotics*				0.303
Yes	39	25 (64.1%)	14 (35.9%)	
No	61	45 (73.8%)	16 (36.2%)	

*Pregnancy status*				0.433
Pregnant	40	26 (65.0%)	14 (35.0%)	
After birth	8	7 (71.2%)	1 (28.8%)	
Not pregnant	52	37 (87.5%)	15 (12.5%)	

*Chronic illnesses*				0.337
Diabetes	7	5 (71.4%)	2 (28.6%)	
Nondiabetic	23	7 (71.2%)	16 (25.8%)	
DVT	3	1 (33.3%)	2 (66.7%)	
Epilepsy	10	6 (42.9%)	4 (57.2%)	
Asthma	11	9 (81.8%)	2 (18.2%)	
TB	6	3 (50.0%)	3 (50.0%)	
Pneumonia	4	3 (75.0%)	1 (25.0%)	

^*∗*^Association of BV (+) with demographic and clinical variables. Significant association between the variable and presence of BV is shown as bold *p* values.

**Table 2:** Prevalence of BV score using Nugent's criteria and HIV infection.

Vaginal flora score	HIV-1 (−)	HIV-1 (+)	*N* value

*BV score*			<0.0001

BV-free, 0–3	18 (62.1%)	11 (37.9%)	
Intermediate, 4–6	12 (30.0%)	28 (70.0%)	
Severe BV, 7–10	9 (29.0%)	22 (71.0%)	

**Table 3:** Association of BV species and HIV infection.

BV score	HIV-1 (−)	HIV-1 (+)	*p* value^*∗*^

*0–3 BV-free*			

Lactobacillus			**0.000**
>25	18 (62.1%)	11 (37.9%)	

Gardnerella vaginalis			0.868
No species	12 (63.2%)	7 (36.8%)	
<25	6 (60.0%)	4 (40.0%)	

Mobiluncus			
No species	17 (63.0%)	10 (37.0%)	0.715
<25	1 (50.0%)	1 (50.0%)	

*4–6 intermediate BV*			

Lactobacillus			0.703
No species	2 (66.7%)	1 (33.3%)	
<25	6 (60.0%)	4 (40.0%)	
>25	20 (74.1%)	7 (25.9%)	

Gardnerella vaginalis			0.655
No species	1 (100%)	0 (0.00%)	
<25	20 (66.7%)	10 (33.3%)	
>25	7 (77.8%)	2 (22.2%)	

Mobiluncus			0.135
No species	7 (100%)	0 (0.00%)	
<25	12 (60.0%)	8 (40.0%)	
>25	9 (69.2%)	4 (30.8%)	

*7–10 severe BV*			

Lactobacillus			0.063
No species	7 (50.0%)	7 (50.0%)	
<25	2 (12.5%)	14 (87.5%)	
>25	0 (0.00%)	1 (100%)	

Gardnerella vaginalis			0.074
<25	6 (46.2%)	7 (53.8%)	
>25	3 (16.7%)	15 (83.3%)	

Mobiluncus			**0.030**
<25	3 (75.0%)	1 (25.0)	
>25	6 (22.2%)	21 (77.8%)	

^*∗*^Association of >25 cells/high microscopic field of BV-associated species with HIV. Significant association between >25 cells/high microscopic field of the species with HIV is shown as bold *p* values.

## Data Availability

Data are available on request from the first author, T. Apalata, via tapalata@wsu.ac.za.

## Supplementary Materials

Supplementary Materials
*S1.* An informed written consent obtained from all the participants. The form was presented to the participants in both English and the indigenous language, isiXhosa. *S2.* Questionnaire used to collect demographic, gynaecological, medical data as well as behavioural risk factors associated with BV from the participants. Demographic data consisted of age, occupation, level of education, and marital status. Gynaecological characteristics included history of gynaecological infections in the past. Behavioural risks included use of contraceptives and use of condoms, whereas medical characteristics included HIV status from available clinical records. The questionnaire is presented in both English and the indigenous language, isiXhosa. ()
