# Supplementary material for: Characterizations of Bacterial Vaginosis among HIV-Positive and HIV-Negative Women in Rural Eastern Cape Province, South Africa
Source: Int J Microbiol. 2021 Jul 21;2021:9913878. doi: 10.1155/2021/9913878 (PMC8321757; doi:10.1155/2021/9913878)
Supplement: Supplementary Materials — S1. An informed written consent obtained from all the participants. The form was presented to the participants in both English and the indigenous language, isiXhosa. S2. Questionnaire used to collect demographic, gynaecological, medical data as well as behavioural risk factors associated with BV from the participants. Demographic data consisted of age, occupation, level of education, and marital status. Gynaecological characteristics included history of gynaecological infections in the past. Behavioural risks included use of contraceptives and use of condoms, whereas medical characteristics included HIV status from available clinical records. The questionnaire is presented in both English and the indigenous language, isiXhosa. () [file 9913878.f1.docx]

**SUPPLEMENTARY MATERIAL**

**S1. CONSENT FORM**

WALTER SISULU UNIVERSITY

FACULTY OF HEALTH SCIENCES

DEPARTMENT OF MEDICAL MICROBIOLOGY

Participant Name / Igama:

Cell Number/ umnxeba:

Consent form for: ***Assessment of Bacterial vaginosis in HIV positive and negative women at Nelson Mandela Academic Hospital.***

By,

Name:

I………………………………………………………………………………….confirm that I have been told what is about to be done to my body and understood the information I’ve been told. On the date of (………/…………/……….) for the above study/ ndiyanqgina ukuba ndizifundile kwaye ndiyaziva inkcukacha zoluhlelo.

I have had the opportunity to consider the information, ask questions and had these answered satisfactory. /ndibenalo ithuba lokucingisisa ngolulwazi ndabuzisise ndadendonela.

|  |
| --- |

I understand that my participation is voluntary and that I am free to withdraw at any time, without giving any reason, without my medical care or legal rights being affected/ndiyazi ukuba Ukunxubelelana kwaloluhlelo kumahala kwaye ndingayeka nanini na gaphandle kwezizathu okanye uchaphazeleleko lwamalungelo wam.

|  |
| --- |

I understand that relevant sections of any data collected during the study may be looked at by responsible individuals from Walter Sisulu University. I give permission to these individuals to have access to my records. Ndinikeza imvume yam kubantu boluluhlu ukuba banganalo inxebelelwano nerecords zam zempilo

|  |
| --- |

I agree to take part in the above research study/ndiyavuma ukuba ndingabandakanyeka koluphando lweziwa apha.

|  |
| --- |

………………………….. ………………………. …………………..

Name of participant Date Signature

Igama Umhla faka isigniture

**S2. QUESTIONNAIRE**

Please mark with an x where appropriate /beka u x kwinkcazelo oyikhethayo

**Demographic characteristics**

1. Name/Igama:

3. How old are you/Uneminyaka emingaphi?

4. Where do you live /Uhlala phi?

1. Are you employed/ingaba uyaphangela na?

| No/hayi |  | Yes/ewe |  |
| --- | --- | --- | --- |

1. Are you married/ingaba utshatile na?

| No/hayi |  | Yes/ewe |  |
| --- | --- | --- | --- |

1. Do you use bubble bath/ingaba uyayisebenzisa isephu xa uhlamba umziba wonke, if yes how often/ukuba kunjalo chaza uba kangaphik?

| No/hayi |  | Yes/ewe  How often/kangaphi? |  |
| --- | --- | --- | --- |

1. Do you smoke/ingaba uyatshaya na?

| No/hayi |  | Yes/ewe |  |
| --- | --- | --- | --- |

**Gynaecological characteristics**

1. History of sexual transmitted infection/amabali ohlalutyo lezifo zocantsi, if yes specify/ukuba kunjalo nika inkcazelo.

| No/hayi |  | Yes /ewe specify which STI/STD/ukuba kunjalo chaza uba sesiphi. |  |
| --- | --- | --- | --- |

1. Are you pregnant or have you ever been pregnant/ingaba ukhulelwe okhanye wakhe wakhulelwa?

| No/hayi |  | Yes?ewe |  |
| --- | --- | --- | --- |

1. Do you have any discharge at the moment/ingaba unayo na idischarge?

|  |  |  |  |
| --- | --- | --- | --- |

1. Colour of discharge/unjani umbala wayo?

|  |  |  |  |
| --- | --- | --- | --- |

1. Is it smelly or bad odor/ingaba inalo na ivumba?

|  |  |  |  |
| --- | --- | --- | --- |

1. How long have you noticed it/lingakanani ixesha unayo?

| Less than 3 days | 3-7 days | 1-2 weeks | months |
| --- | --- | --- | --- |

1. Do you have any pelvic pains/ingaba awuqaqanjelwa na ngamazantsi esisu?

| Yes/ewe |  | No/hayi |  |
| --- | --- | --- | --- |

**Behavioural factors**

1. Do you use condoms/Ingaba uyazikhusendela xa usabelana ngesondo?

| No/hayi |  | Yes/ewe |  |
| --- | --- | --- | --- |

1. How many sexual partners do you have/bangaphi abantu obelana nabo ngosondo?

| one |  | More than one |  |
| --- | --- | --- | --- |

1. Do you use contraceptives/imgaba uyacwangcisa ukuzikhusela uba ungakhulelwa na?

| No/hayi |  | Yes/ewe |  |
| --- | --- | --- | --- |

1. If yes,which contraceptive do you use/leliphi ihlobo lokucwangcisa olusebenzisayo?

|  |
| --- |

1. Have you ever tested for HIV test? If yes what is your status/ wawukhe wayihlolela intsholongwana kagawulayo?ukuba kunjani sithini isimo sakho?

| No/hayi |  | Yes/ewe  Status/isimo sakho: |  |
| --- | --- | --- | --- |

**Medical characteristics**

1. Do you have any diagnosed health problems /ingaba sikhona na isigulo onaso okanye owakhe wanaso?

| Yes/ewe |  | No/hayi |  |
| --- | --- | --- | --- |
| Diagnosed health problem/isgulo | |  | |
| How long have you had this health problem/ lide kangakanani ixesha unaso? | |  | |

1. Are you taking any pills or antibiotics/ingaba akhona amachiza owasebenzisayo?

| Yes,specify/  ewe,nika inkcazelo ukuba ngawaphi: |  | No/hayi |  |
| --- | --- | --- | --- |
